# Evidence for a Genotype–Phenotype Correlation in Patients with Pathogenic *GLUT2* (*SLC2A2*) Variants

**DOI:** 10.3390/genes12111785

**Published:** 2021-11-10

**Authors:** Sarah C. Grünert, Anke Schumann, Federico Baronio, Konstantinos Tsiakas, Simona Murko, Ute Spiekerkoetter, René Santer

**Affiliations:** 1 Department of General Pediatrics, Adolescent Medicine and Neonatology, Medical Center—University of Freiburg, 79106 Freiburg, Germany; anke.schumann@uniklinik-freiburg.de (A.S.); ute.spiekerkoetter@uniklinik-freiburg.de (U.S.); 2 Pediatric Unit, Department of Medical and Surgical Sciences, Regional Center for Expanded Newborn Screening, S. Orsola—Malpighi University Hospital, 40138 Bologna, Italy; federico.baronio@aosp.bo.it; 3 Department of Pediatrics, University Medical Center Eppendorf, 20246 Hamburg, Germany; ktsiakas@uke.de (K.T.); s.murko@uke.de (S.M.); r.santer@uke.de (R.S.)

**Keywords:** Fanconi-Bickel syndrome, genotype-phenotype correlation, *GLUT2*, *SLC2A2*, glycogen storage disease, glucosuria

## Abstract

Fanconi-Bickel syndrome (FBS) is a very rare but distinct clinical entity with the combined features of hepatic glycogen storage disease, generalized proximal renal tubular dysfunction with disproportionately severe glucosuria, and impaired galactose tolerance. Here, we report five cases (out of 93 diagnosed in our lab) with pathogenic variants on both *GLUT2* (*SLC2A2*) alleles. They come from 3 families and presented with an exceptionally mild clinical course. This course was correlated to data from old and most recent expression and transport studies in *Xenopus* oocytes. *GLUT2* genotype in patients 1 and 2 was p.[153_4delLI];[P417R] with the first variant exhibiting normal membrane expression and partially retained transport activity (5.8%) for 2-deoxyglucose. In patient 3, the very first *GLUT2* variant ever detected (p.V197I) was found, but for the first time it was present in a patient in the homozygous state. This variant had also shown unaffected membrane expression and remarkable residual activity (8%). The genotype in patient 4, p.[153_4delLI];[(E440A)], again included the 2-amino-acid deletion with residual transporter function, and patient 5 is the first found to be homozygous for this variant. Our results provide further evidence for a genotype-phenotype correlation in patients with *GLUT2* variants; non-functional variants result in the full picture of FBS while dysfunctional variants may result in milder presentations, even glucosuria only, without other typical signs of FBS.

## 1. Introduction

Fanconi-Bickel syndrome (FBS, OMIM #227810) is a rare autosomal recessive disorder of carbohydrate metabolism caused by pathogenic variants in the *GLUT2* (*SLC2A2*) gene [1]. *GLUT2* encodes the glucose transporter-2 protein, a member of the facilitative glucose transporter family, which is mainly expressed in liver, pancreas, intestine and kidney. Since the first description in a Swiss boy in 1949 [2], around 90 FBS cases have appeared in the literature [3]. FBS has been defined as a hepatic glycogen storage disorder with hepato- and nephromegaly from hepatorenal glycogen accumulation, impaired glucose release during fasting, and a propensity to hypoglycemia. This is typically combined with proximal renal tubular dysfunction as a result of the accumulation of glycogen and free glucose within renal tubular cells and causes the renal Fanconi syndrome including hypophosphatemic rickets. Impaired utilization of both glucose and galactose results in postprandial hyperglycemia and galactosemia. Finally, severely stunted growth has its cause in several interacting factors [4].

Since our first description of the underlying genetic defect of this well-defined clinical entity [1], we have diagnosed 93 patients with biallelic variants of *GLUT2*. In this report we summarize clinical and genetic findings of 5 of these patients with a very mild and exceptional presentation, we relate their genetic findings to published functional data and provide further evidence for a genotype-phenotype correlation in patients with a variable degree of *GLUT2* deficiency.

## 2. Materials and Methods

Cases with an atypical clinical presentation were retrieved from our internal database established 1997–2021, currently containing 93 individuals with biallelic *GLUT2* variants. The *GLUT2* sequencing method has changed over the years from a protocol for ^33^P-labeled terminators provided with the Thermo-Sequenase cycle sequencing kit (Amersham/Buchler, Wenden, Germany) [5] to a standard sequencing technique using the ABI BigDye Terminator Sequencing Kit (Applied Biosystems, Darmstadt, Germany) and an automated capillary sequencer (ABI 3500; Applied Biosystems, Darmstadt, Germany). Sequence electropherograms were analyzed using the Sequence Pilot software (JSI Medical Systems, Ettenheim, Germany) and ENST00000314251.8 (NM_000340.1) as the transcript reference sequence.

## 3. Patients and Results

**Figure 1:**
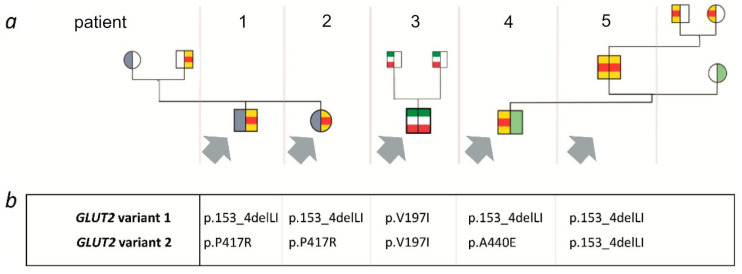
(**a**) Pedigree of affected families; (**b**) *GLUT2* (*SLC2A2*) genotype in patients 1–5.

A summary of clinical, laboratory and genetic findings of all 5 patients is shown in Table 1 and Figure 1.

### 3.1. Family 1

Patients 1 and 2 are siblings born to non-consanguineous German parents. Clinical and laboratory findings of both patients have previously been reported [6]. Patient 1, a 19-year-old boy at the time of this report, was diagnosed at the age of 9 months when he presented with failure to thrive. *GLUT2* deficiency was suspected because of elevated transaminases, glucosuria, mild tubular proteinuria, and an elevated postprandial galactose concentration in blood. Liver size was normal. Targeted sequencing of the *GLUT2* gene revealed compound heterozygosity for a 6-bp in frame deletion in exon 4 (c.457_462delCTTATA; p.153_4delLI) and a missense variant in exon 10 (c.1250C>G; p.P417R).

His younger sister (now 14 years old) was diagnosed within the first month of life. Similar to her brother, she presented with an elevated blood galactose concentration, glucosuria and mild proteinuria. Molecular genetic analysis was performed and the girl was found to be compound heterozygous for the same *GLUT2* variants as her brother. Remarkably, newborn screening results of both children were normal with respect to galactose concentrations.

In both children, a diet restricted in free glucose and galactose was introduced with the recommendation of frequent feedings and the avoidance of fasting. Uncooked corn starch (0.5 g/kg bw/d) was given at bedtime for two years. On this dietary regimen, the metabolic condition of both children was very stable. Glucose and galactose concentrations were within the normal range, and transaminases normalized. Hepatomegaly, nephromegaly and hypophosphatemic rickets have never been observed. Glucosuria was a constant finding, while proteinuria remained very mild and was even absent in some urine samples. No decline in kidney function has been observed. Both children showed normal growth and psychomotor development.

### 3.2. Family 2

Patient 3 has been published in abstract form by Baronio et al. in 2012 [7]. In this 17-year-old Italian boy glucosuria and galactosuria was an incidental finding at the age of 8 months. Due to postprandial hyperglycemia he was treated with insulin glargine for several years in addition to a galactose-restricted diet. Thereafter, an oral and intravenous glucose tolerance test did not show any severe abnormality. Liver and kidney size were normal as documented by abdominal ultrasound. A urine examination revealed mild glucosuria without any other signs of tubulopathy. Osteopenia was diagnosed by DXA scan. His physical growth and psychomotor development were normal. Targeted sequencing of the *GLUT2* gene revealed homozygosity for a previously reported variant (c.589G>A; p.V197I) with heterozygosity in both parents.

### 3.3. Family 3

The 2-year-old boy is the first child of non-consanguineous German parents not knowingly related to patients 1 and 2 but from the same geographic area. He was delivered spontaneously after an unremarkable pregnancy. Newborn screening revealed a massive elevation of blood galactose (2500 µmol/L) and galactose-1-phosphate (13.8 mg/dL) concentrations. When clinically evaluated, the child was asymptomatic and showed no signs of hepatic dysfunction. Transaminases, bilirubin and coagulation studies were normal. Due to a suspected disorder of galactose metabolism, he was put on a galactose-free formula. Galactose-1-phosphate uridyltransferase (GALT) deficiency, uridine diphosphate galactose 4-epimerase (GALE) deficiency and galactokinase (GALK) deficiency were excluded by enzymatic and/or genetic investigations. Plasma biotinidase activity, known to be elevated in patients with hepatic glycogen storage diseases, was 80% of normal mean. No hepatomegaly was present. Urine analysis revealed mild glucosuria (2500 mg/L) despite normal blood glucose concentrations, generalized hyperaminoaciduria, but only very mild proteinuria. Therefore, targeted analysis of the *GLUT2* gene was performed with the result of compound heterozygosity for the previously described 6-bp deletion (c.457_462delCTTATA; p.153_4delLI) and a missense variant (c.1319C>A; p.(A440E)).

On a galactose-restricted diet the patient showed normal growth and development. At age 18 months, his body length was on the 87th centile, and his weight on the 91th centile.

His transaminases have never been elevated and typical clinical symptoms of FBS including hepatomegaly or rickets have not been observed. He displays variable but at times significant glucosuria (250–2350 mg/dL) with only mild proteinuria and hyperaminoaciduria.

Patient 5 is the 29-year-old father of patient 4. At the age of 10 months, glucosuria and galactosuria were first detected during a febrile infection. Defects of galactose metabolism as well as a portocaval shunt were excluded. A mild restriction of dairy products was recommended. A follow-up investigation at 7 years showed only mild glucosuria. His growth and puberty were normal, his adult height is 183 cm. Targeted genetic testing for *GLUT2* variants was performed after the diagnosis had been made in his son and revealed homozygosity for the aforementioned 6-bp deletion (c.457_462delCTTATA; p.153_4delLI). Heterozygosity for this sequence aberration was confirmed in both of his parents who were not aware of consanguinity.

When assessed at our metabolic center, he did not report any health issues. He was on an unrestricted diet including dairy products. He has never experienced episodes of hypoglycemia, neither hepatomegaly nor signs or symptoms of rickets were detected. Laboratory testing showed normal transaminases and a normal HbA1c. The only laboratory finding was isolated glucosuria (950 mg/dL) without proteinuria or hyperaminoaciduria.

## 4. Discussion

We report a series of 5 cases from 3 families with biallelic *GLUT2* variants with an exceptionally mild clinical course when compared to our total cohort of 93 patients (Figure 1). The main clinical feature of these cases was glucosuria and/or galactosuria/galactosemia while other hallmarks of typical FBS were lacking. Although FBS was originally classified as a hepatic glycogen storage disease, none of these 5 patients had hepato- or nephromegaly or fasting hypoglycemia, and all 5 individuals showed normal growth. If present at all, proteinuria was very mild, and no patient displayed hyperphosphaturia or rickets.

Some patients with FBS can be detected by abnormal newborn screening for galactosemia due to their impaired hepatic uptake of galactose that may results in an elevated blood galactose concentration and even cataract [8]. It is of note, that only 1 of our 5 patients with a mildly impaired *GLUT2* function displayed an elevated galactose concentration in newborn screening, although all of them had an elevated galactose concentration in blood during the diagnostic work-up, probably due to increasing lactose intake with time. Interestingly, patient 5 was found to have combined glucosuria and galactosuria as well as elevated galactose levels in blood in early childhood, but galactosemia was no longer present in adulthood even on an unrestricted intake of dairy products.

This cohort of mildly affected individuals with *GLUT2* variants demonstrates that individuals with residual activity of the *GLUT2* protein may present with isolated glucosuria only and it shows that impaired *GLUT2* function must be considered in the differential diagnosis of renal glucosuria even in the absence of other typical signs and symptoms of FBS. Aminoaciduria may be present in such cases but it does not necessarily reflect tubulopathy. As in SGLT2 deficiency it can simply be the result of solvent drag secondary to the high amounts of unabsorbed glucose [9].

To date, 46 *GLUT2* variants associated with FBS have been identified, including missense, nonsense, in-frame indels (insertions/deletions), frameshift indels, and splice site variants [3]. Four of our patients share the same 6-bp/2-amino-acid deletion (c.457_462delCTTATA; p.153_4delLI), thereof 3 in compound heterozygosity and one in homozygosity. Data retrieval from the gnomAD database (14 October 2021) show an allele frequency for this variant of 22 among 281,014 alleles tested. This is rare but due to the number of cases observed in our study, we speculate that it is higher in our geographic area, the south-western part of Germany. Enogieru et al. have recently published expression and transport studies of 17 *GLUT2* variants in *Xenopus* oocytes [3]. In this study, the in-frame deletion c.457_462delCTTATA was the only variant associated with clinical symptoms that was shown to have partially retained transport activity for 2-deoxyglucose (5.8% of wildtype). Membrane expression of the mutant *GLUT2* protein, p.Leu153_Ile154del, was comparable to wild type. The 2-amino-acid deletion is located between the transmembrane helices 3 and 4 of the *GLUT2* protein. Information on possible conformational changes of the protein caused by this genetic variant is not available.

Patients 1 and 2 are compound heterozygotes for the variants p.Leu153_Ile154del and p.Pro417Arg. The p.Pro417Arg variant was never before seen in an FBS patient but was also functionally studied by Enogieru et al. [3] and shown to have no activity. In patient 4, the p.Leu153_Ile154del variant was found in combination with *GLUT2* c.1319C>A; p.(A440E) which is also a novel variant and not previously reported in FBS. This missense variant is classified as pathogenic by several prediction programs and has only been identified once in heterozygosity in around 250,000 exomes. To our knowledge, functional data are not available for this variant. Patient 5 is the first patient found to be homozygous for the p.Leu153_Ile154del variant. He is the oldest individual in this case series and lives an unimpaired life.

Patient 3 is homozygous for the *GLUT2* c.589G>A; p.V197I variant. This is the very first variant ever reported for *GLUT2*. It was originally identified in the heterozygous state in an African-American woman with type 2 diabetes mellitus [10] and it has repeatedly been discussed whether this was an incidental finding or causally related to diabetes. It is still listed in gnomAD as a variant of uncertain significance with an allele frequency of 13 in 281,780 (data retrieval 14 October 2021). Our case is the first homozygous individual carrying this variant which confirms that it definitely affects glucose transport. Due to this historical aspect, however, results from expression studies in *Xenopus* oocytes had already been reported by Mueckler et al. [11] in 1994. The valine to isoleucine change in position 197 was shown to reduce transport activity of the *GLUT2* protein but only to about 8% of wild type activity and membrane expression of the *GLUT2* p.V197I protein was unaffected as shown by Western blots [11].

Although it is clear also from this case that a missense variant with residual activity results in clinically relevant impairment of glucose transport, the question of the role of heterozygosity for *GLUT2* missense variants in diabetes remains open. Several heterozygous individuals with type-2 diabetes, including the mother of patient 3 with heterozygosity for the *GLUT2* p.V197L variant, were indeed detected in this family; on the other hand, the heterozygous father showed a completely normal oral glucose tolerance test (oGTT). This means that heterozygosity for *GLUT2* variants might be a risk factor for the development of type-2 diabetes, as demonstrated in several studies [12,13,14] but other predisposing factors are required.

Enogieru et al. [3] have already hypothesized that the residual activity found in the p.Leu153_Ile154del transport protein, albeit minimal, seems to be sufficient to rescue the severe phenotype that is commonly associated with FBS. Our data support this hypothesis and provide further evidence for a genotype-phenotype correlation in this rare disease. We therefore suggest to use the general term *GLUT2* deficiency for cases like ours and to restrict the term FBS to those with the original typical syndromic presentation.

## 5. Conclusions

Our results provide further evidence for a genotype-phenotype correlation in patients with *GLUT2* variants. Non-functional variants result in the full picture of FBS while dysfunctional variants depending on severity may result in a markedly attenuated presentation recognizable only by impaired sugar transport in the kidney. Therefore, *GLUT2* deficiency has to be included in the differential diagnosis of glucosuria even when other typical clinical signs of FBS are lacking and it may be mistaken for renal glucosuria caused by genetic variants of *SGLT2* (or *MAP17*). Aminoaciduria may not distinguish the two conditions but concomitant galactosuria discriminates renal glucoseuria caused by *GLUT2* deficiency from SGLT2 deficiency and may guide genetic testing.

## Figures and Tables

**Table 1:** Typical features of Fanconi-Bickel syndrome (FBS) and summary of clinical findings in patient 1–5. (* previously reported [6]); oGTT, oral glucose tolerance test.

Typical Findings in FBS	Patient 1 * (19 y)	Patient 2 * (13 y)	Patient 3 (17 y)	Patient 4 (2 y)	Patient 5 (29 y)
Hepatomegaly	absent	absent	absent	absent	absent
Fasting hypoglycemia	absent	absent	absent	absent	absent
Postprandial hyperglycemia	normal oGTT but intermittend postprandial hyperglycemia at time of diagnosis, HbA1c within normal range	absent HbA1c within normal range	normal oGTT but intermittend postprandial hyperglycemia	absent HbA1c within normal range	absent HbA1c within normal range
Galactose intolerance	yes	yes	yes	yes	yes galactosemia and galactosuria during childhood, only galactosuria in adulthood
Nephromegaly	absent	absent	absent	absent	absent
Proteinuria	very mild or absent	very mild or absent	absent	very mild	absent
Glucosuria massive (often >100 g/1.73 m^2^/d)	yes no 23.6	yes no 34.1	yes no 1.0	yes no 11–23 (g/L)	yes no 5 (g/L)
Hyperaminoaciduria	absent	absent	absent	yes	absent
Hyperphosphaturia	absent	absent	absent	absent	absent
Rickets	absent	absent	absent	absent	absent

## Data Availability

The data presented in this study are available on request from the corresponding author.
